# Ligation of the Intersphincteric Fistula Tract as an Emergency Treatment for Cryptoglandular Anal Fistula

**DOI:** 10.21315/mjms2024.31.1.5

**Published:** 2024-02-28

**Authors:** Diana Melissa Dualim, Michael Pak-Kai Wong, Siti Mayuha Rusli, Abdel Latif Khalifa Elnaim Ali, Ismail Sagap

**Affiliations:** 1 Colorectal Unit, Department of Surgery, Faculty of Medicine, Universiti Kebangsaan Malaysia, Kuala Lumpur, Malaysia; 2 School of Medical Sciences and Hospital Universiti Sains Malaysia, Universiti Sains Malaysia, Kelantan, Malaysia; 3 Department of Surgery, Kassala Police Hospital, Kassala, Sudan

**Keywords:** anal fistula, ligation, abscess, emergency treatment, elective surgical procedures

## Abstract

**Introduction:**

Ligation of the intersphincteric fistula tract (LIFT) is a sphincter-preserving procedure for treating anal fistula of cryptoglandular origin. Our prospective study aimed to determine the postoperative outcomes of patients undergoing LIFT in emergency and elective settings.

**Methods:**

This was a single-centre prospective observational study of the LIFT procedure for the treatment of anal fistulas. The differences in the 6-month postoperative outcomes between the emergency and elective procedures were analysed, including the healing rate, healing time, recurrence rate, recurrence time, postoperative complications and length of hospital stay.

**Results:**

Twenty-two patients were recruited for this study: 11 patients underwent LIFT as an emergency procedure (EM-LIFT), while the others underwent LIFT as an elective procedure (EL-LIFT). The healing rate for the EM-LIFT group was 90.9% (*n =* 10), with a median healing time of 2 months (range 0.5–4). For the EL-LIFT group, the healing rate was 100% (*n =* 11), with the same median healing time of 2 months (range 0.5–4). Two of the patients in the EM-LIFT group developed recurrence, with a median recurrence time of 5 months (range 4–6) and three developed recurrence in the EL-LIFT group, with the same median recurrence time of 5 months (range 4–6). There were minor postoperative complications of pain and subcutaneous infection, with no faecal incontinence. There was no statistically significant difference in postoperative outcomes between the groups.

**Conclusion:**

EM-LIFT is a feasible and safe primary procedure for active cryptoglandular-type anal fistulas.

## Introduction

An anal fistula is an abnormal communication between the anal canal and the perianal skin resulting from a chronic inflammatory process (1). The classical presentations are cyclical perianal pain with chronic intermittent purulent drainage. Anal fistulas account for about 65% of patients with perianal abscesses (2). In the acute phase, the condition is usually associated with purulent discharge after rupture and spontaneous decompression (2). The goals of successful anal fistula treatments are an early healing rate, a low recurrence rate and preservation of anal continence (2, 3). The primary modality of treatment is surgery, which eliminates septic foci from the associated epithelialised fistula.

In 2007, a novel total sphincter-preserving technique, referred to as ligation of the intersphincteric fistula tract (LIFT), was introduced for the treatment of anal fistula (4). The initial outcome demonstrated a promising result, with a primary healing rate of 94.4% and negligible incontinence (4). The principles of this technique are removal of the infected cryptoglandular tissue through the intersphincteric approach and closure of the internal opening within the intersphincteric space, which disconnects the continuous faecal material from exiting the fistula tract through the external perianal opening. This results in complete healing of the anal fistula while maintaining the integrity of the anal sphincter (2, 5).

To date, this is the first local study exploring the feasibility of LIFT as an emergency procedure (EM-LIFT) for active anal fistulas. Active anal fistula presenting with pain and continuous purulent discharge has conventionally been treated with seton insertion and followed by a scheduled LIFT procedure within 6–12 weeks. This two-stage procedure allows the tract to mature and the cavity to drain, which facilitates ligation at a later date (6). However, this technique has a longer healing time that varies from 3 months–9 months (3). LIFT has been found to be associated with a reasonable healing rate and complete preservation of the anal sphincter (3, 7, 8). Other sphincter-preserving procedures, such as fibrin glue, fistula plug or advancement flap, have been shown to have less favourable success rates on longer-term follow-up (9–13).

This study aimed to examine and compare the feasibility and postoperative outcomes of performing LIFT in an emergency setting versus a conventional elective setting (EL-LIFT).

## Methods

A single-centre prospective observational study was conducted on patients with cryptoglandular anal fistulas who underwent LIFT procedures at the Department of Surgery, Universiti Kebangsaan Malaysia, from 1 September 2017 to 31 September 2019. The inclusion criteria were an age of more than 18 years old and anal fistula of cryptoglandular origin presenting for the first time or recurrent presentation with or without previous surgical intervention. Patients with underlying anorectal malignancy, inflammatory bowel disease, HIV or tuberculosis were excluded from the study. Informed consent for the study was obtained once the decision for EM-LIFT or EL-LIFT was made at the discretion of the colorectal surgeon based on the clinical presentation.

The emergency setting in our study was defined as an active anal fistula presenting with pain and purulent discharge that required a LIFT procedure within the same admission under an emergency operation list. The elective setting was defined as an anal fistula that had either been previously drained by seton insertion or that presented without active purulent discharge and was scheduled for a LIFT procedure on the elective operation list.

A complex anal fistula was defined as a recurrent anal fistula or the presence of a secondary tract. Healing was defined as the closure of the fistula tract, which included the closure of the external opening and the intersphincteric wound. Recurrence referred to persistent purulent discharge from the external opening or the intersphincteric wound after the index surgery wound healed. Persistent purulent discharge from the external wound and/or intersphincteric wound postoperatively was considered a non-healing wound (3).

The participant selection process started with a complete history and physical examination, including a per-rectal examination to inspect the presence of an external opening. Patients who presented with perianal discharge and abscesses were recruited for EM-LIFT on the same day. In contrast, anal fistulas without persistent perianal discharge and abscess were recruited for EL-LIFT, which was scheduled within 6 weeks to 12 weeks of presentation. The participants who met the selection criteria were given a patient information sheet and informed consent was obtained by explaining the indication of the LIFT procedure, including the operative steps, risks and complications. The LIFT procedure was performed by colorectal surgeons from our unit.

A preoperative rectal enaema was given to each patient. After regional anaesthesia, the patient was positioned in an extended Lloyd-Davies position using Yellofin stirrups. Intraoperative examination under anaesthesia with digital rectal examination and endoanal ultrasound was performed to delineate the anatomy of the anal fistula. The decision to proceed with LIFT was made intraoperatively by the surgeon. The details of the technique were identical to those initially proposed by Rojanasakul (4). In the proposed technique, the essential steps were a curve-linear incision at the intersphincteric groove, identification of the intersphincteric tract, ligation of the intersphincteric tract close to the internal opening and removal of the intersphincteric tract via curettage. The defect at the external sphincter muscle was sutured using an absorbable suture (4). Both groups of patients received a dose of intravenous antibiotics (cefoperazone and metronidazole) during induction.

All patients were only allowed to discharge after adequate analgaesia was achieved and the sepsis had resolved. Each discharged patient received oral analgaesia (paracetamol and tramadol), a stool softener (lactulose) and oral antibiotics (cefuroxime and metronidazole) to be taken for 7 days after the procedure. They were taught to perform a sitz bath three times a day, starting on the first postoperative day. They were all followed up for 6 months: first at 2 weeks after the procedure and subsequently in the second, fourth and sixth months. During each visit, wound healing, recurrence status, continence status and other postoperative complications were evaluated.

Patients with persistent perianal discharge were advised to continue the regular sitz bath and 2 weekly follow-ups for wound review. Patients with recurrence were reassessed with either an endoanal ultrasound or an MRI pelvis. Preoperative and postoperative anal continence were assessed using a validated Wexner’s Incontinence Scale (WIS) (14). Patients who did not present on follow-up were contacted by telephone and symptomatic patients were offered early walk-in appointments for immediate evaluation.

The sample size calculation was based on the frequency of a population size of 20 (for finite population correction factor, *N*), with the hypothesised recurrence rate as the outcome factor for the population (*p*), which was determined to be 5.9% from a previous study (7). The confidence limit (*d*) was set at 5%, with a design effect of one used to estimate the sample size. The required sample size for this study was determined to be 17 patients and after considering a 20% dropout rate, the final sample size required was 20 (*N* = all patients who underwent LIFT at the study location). For this study, we managed to recruit 27 patients; however, only 22 patients underwent LIFT procedures. Subsequently, a sub-analysis was conducted to compare the outcomes of LIFT performed in acute settings (EM-LIFT) and LIFT performed in elective settings. Due to the limited study time and sample size, we managed to recruit only 11 patients in each arm.

Statistical analyses were performed using IBM SPSS version 22.0. The results were expressed as medians (interquartile range or IQR) and absolute values with percentages. The Mann-Whitney U test was applied to compare the continuous variables, while frequency distributions were used to describe categorical variables and are presented as *n* (%). The comparison of categorical variables was done with Fisher’s exact test and the statistically significant *P*-value was set at 0.05.

## Results

A total of 27 patients were recruited for this study. Five patients were excluded from the study because they did not meet the inclusion criteria. All patients had external openings, including the excluded patients; two had superficial anal fistulas, for which we performed a fistulotomy. The other three excluded patients had no mature fistula tracts for ligation. Therefore, we performed drainage and seton insertion for two patients and one incision and drainage due to no identifiable internal opening.

**Figure 1:**
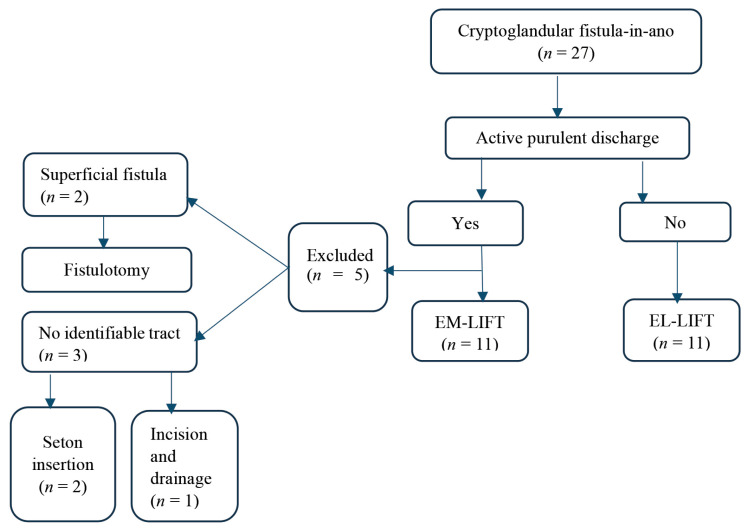
Recruitment and selection

The remaining 22 patients who fulfilled the inclusion criteria were equally divided into 11 participants in each arm of the study, EM-LIFT or EL-LIFT, upon the discretion of the treating surgeon (Figure 1).

The median age of the patients was 35 years old (IQR = 10; range 29–64) in the EM-LIFT group and 40 years old (range 28–65) in the EL-LIFT group. The majority of the patients were male. Three patients (27%) from each group had underlying diabetes mellitus. Two of the patients (18%) in the EM-LIFT group and three (27%) in the EL-LIFT group were obese (Table 1).

In the EM-LIFT group, seven patients (63%) had recurrent cases. Of these, four (36.6%) had undergone previous surgical intervention (incision and drainage with seton insertion). For the EL-LIFT group, five (45.5%) were recurrent cases at presentation, with all having had previous surgical intervention (in the form of seton insertion). Two patients (18.8%) were in sepsis in the EM-LIFT group but none were in the EL-LIFT group. Seven patients (63.6%) in the EM-LIFT group had complex fistulas, four had previous surgical interventions and another three had secondary fistula tracts. Of the patients in the EL-LIFT group, eight (72.2%) had complex fistulas, three had previous surgical interventions, three had secondary fistula tracts and two had secondary fistula tracts with previous surgical interventions (Table 2).

The median operative time was 45 min (range 30–110) in the EM-LIFT group and 60 min (range 10–140) in the EL-LIFT group, but this difference was not statistically significant. The median hospital stay for the EM-LIFT group was 2 days (range 1–3), while for the EL-LIFT group, it was 0 day (range 0–1). Among the EM-LIFT patients, wound healing was observed in 10 patients (90.9%), with a median healing time of 2 months (range 0.5–4), while in the EL-LIFT group, all 11 patients (100%) had healed wounds, with a median healing time of 2 months (range 0.5–4; *P* = 0.770). Recurrence was seen in 2 out of 11 patients in the EM-LIFT group, with a median recurrence time of 5 months (range 4–6), while three out of 11 patients developed recurrence in the EL-LIFT group, with a median recurrence time of 5 months (range 4–6; Table 3).

There was no significant difference in complications noted between the arms. The complications reported included non-healing wounds, perianal pain and subcutaneous infection. Perianal pain was adequately controlled in nine patients (81.8%) in the EM-LIFT arm and seven (63.6%) in the EL-LIFT arm. Two patients in the EM-LIFT arm and four patients in the EL-LIFT arm had their pain completely resolved at the 2-month follow-up. A subcutaneous infection developed in one patient from each group, which healed with conservative treatment after 2 weeks. None of the patients in either group developed anal fissure, local haemorrhage or faecal incontinence (Table 3).

## Discussion

The treatment of anal fistula is mainly surgical to eliminate septic foci and any associated epithelialised fistula tract; hence, the aim is to cure the disease with preservation of faecal continence and to prevent recurrence (2, 3). Surgical treatment of a perianal fistula can be divided into sphincter-sacrificing and sphincter-sparing methods. Sphincter-sparing methods include fistulotomy, fibrin glue application, endorectal advancement flap, video-assisted technique (VAAFT) and LIFT techniques (5). All of these methods have varied healing rates and negligible incontinence rates. In contrast, sphincter-sacrificing techniques, either with or without immediate repair, have a high healing rate but a high postoperative incontinence rate. The impairment of continence has a negative effect on quality of life, so sphincter-sparing methods are now preferred (3).

In 2007, Rojanasakul (4) introduced LIFT with a promising healing rate of 94%, as the procedure is simple, safe and effective, with a negligible risk of incontinence. It was also found that cases of treatment failure or recurrence might have been related to inadequate management of sources of infection or the presence of remnant fistula tract (2, 3). The principle of the LIFT procedure is to excise and ligate the intersphincteric tract, which impedes the entry of faecal particles into the fistula and at the same time, eliminates the intersphincteric septic focus. This allows for complete healing and closure of the fistula tract and postoperative preservation of continence (2, 5). LIFT was chosen as the sphincter-sparing technique in this study because the procedure is easily reproducible, with a shallow learning curve and applicability in both recurrent and complex cases. Draining seton is also a simple technique but it has a lengthy healing time, varying from about 3 months–9 months and often requires a multi-staged procedure to achieve healing (3). Most of the published data on LIFT exclude patients with anorectal abscesses, as such procedures are performed under elective settings (1). In contrast, this study explores the outcome of performing LIFT in an active anal fistula with acute purulent discharge in an emergency setting.

The main determinant for a successful LIFT procedure is a healed wound with no recurrence or preservation of sphincter function. In previous studies on LIFT, the average wound healing time ranged from 4 weeks–8 weeks, with healing rates varying between 57% and 94% (15). LIFT performed in emergency settings in this study was successful without resulting in functional impairment, with a healing rate of 90.9% (*n* = 10) and a median healing time of 2 months (range 0.5–4), which is similar to the outcomes of those procedures performed electively. These results show that LIFT is safe and feasible to conduct as an index procedure during the acute purulent phase of an active anal fistula. Hence, we can avoid the staged procedure, which is frequently practiced as a seton-first-LIFT-later approach (16). Many studies against the use of long-standing seton placement before a LIFT procedure suggest that the seton may lead to overt inflammation and granulation at the internal sphincter and the intersphincteric space, which results in technical difficulty when delineating and dissecting the fistula tract during the LIFT procedure (6).

The cases of treatment failure and recurrence after LIFT described in our study were similar to those detailed by Tan et al. (17), who discussed treatment failure as a non-healing surgical wound or fistula and recurrence as reappearance of the fistula after initial healing. One patient in the EM-LIFT arm had a non-healing intersphincteric wound but a closed external anal fistula opening. Internal and external openings were located in the 3 o’clock region during the first operation. Reassessment during the second operation with endoanal ultrasound showed the presence of another internal opening in the 6 o’clock region, which was not detected during the first operation. This patient was treated with a draining seton and subsequently underwent EL-LIFT. The anal fistula eventually healed completely after 6 months with preserved continence.

The recurrence rate for LIFT has been reported elsewhere to be between 5.6% and 58%, with a median recurrence time of 2 months– 8 months (1). The recurrence rate in this study was 22.7%, which included two patients from the EM-LIFT group and three patients from the EL-LIFT group. The two patients in the EM-LIFT group had recurrences at 4 months and 6 months. These two EM-LIFT patients had the initial external opening closed. However, a new external opening occurred at the intersphincteric incision site, henceforth forming a low intersphincteric anal fistula that was treated successfully with fistulotomy. The recurrence rate in the present study was consistent with those of other studies with reported median recurrence times of 2 months–8 months (1, 17–20).

Other postoperative complications of LIFT were local haemorrhage (4%), anal fissure (< 1%), persistent pain (< 1%), subcutaneous infection (< 1%) and faecal incontinence (1, 19, 21). There have yet to be data reported on the postoperative complication rate of EM-LIFT. None of the patients in our study, including those who had recurrence, complained of incontinence at the six-month follow-up. This result shows that LIFT in an emergency setting is not associated with a higher risk of sphincter injury causing faecal incontinence, as previously hypothesised (1, 19, 21). While persistent pain was reported in two patients in the EM-LIFT group and four patients in the EL-LIFT group, it was completely resolved in both cases after 2 months. Subcutaneous or superficial infection was present in both groups of patients, but it completely healed after 2 weeks of antibiotic treatment. Finally, local haemorrhage was reported in 4% of patients in other studies; however, no patients reported such an occurrence in either group of our cohort (19, 22, 23).

Furthermore, there was no difference in the median operating time between EM-LIFT and EL-LIFT. The median duration of both groups was comparable with the reported EL-LIFT median duration of 67.5 min (18). The EM-LIFT procedure had a similar difficulty index to EL-LIFT. In addition, the patients from the EL-LIFT group had more complex fistula anatomy compared to the EM-LIFT group; while this explained the longer median operative time in EL-LIFT, the difference was not statistically significant.

It should also be noted that the hospital stay was statistically significantly longer in the EM-LIFT group compared to the EL-LIFT group; this is because the majority of the patients were in sepsis (54.5%) and required longer recuperation periods before discharge.

In general, this study shows that LIFT can play an important role in the management of anal fistulas in emergency settings. We also showed that performing LIFT in such circumstances, even in the presence of active purulent discharge and collection, yields a similar outcome compared to the staged LIFT procedure.

## Conclusion

The EM-LIFT procedure yielded the same outcome as the electively performed procedure. Therefore, the LIFT procedure is a feasible and safe index operation for the emergency treatment of cryptoglandular-type anal fistulas with local sepsis.

### Limitation of the Study

Our small sample size limited us from performing a more extensive statistical comparison between the two groups. Specifically, such collection was limited by slow recruitment during the study period. The estimated sample size was 20, with 20% dropout consideration. For our study, we recruited 27 patients, with only 22 patients undergoing LIFT procedures. This shortcoming was due to the limited study period and protracted recruitment process. The calculated power of the study was 60%.

Our study was set up to explore short-term outcomes, hence the short follow-up period. We suggest that future studies extend their duration of study to 24 months to evaluate the long-term outcomes and recurrences.

There may also be the presence of selection bias, since the allocation was based on the discretion of the treating physician. Although it was not statistically different, we are inclined against performing LIFT for complex anal fistulas in elective settings. This should be at the discretion of the surgeon, as they may feel it is safe to perform staged LIFT in this group of patients.

### Strengths of the Study

Both groups had similar demographic distributions and complexities with respect to anal fistula. In addition, this is the first study designed to observe and explore the feasibility of EM-LIFT for the treatment of acute anal fistula. This study should encourage a larger design or randomised controlled trial for EM-LIFT.

## Figures and Tables

**Table 1:** Sociodemographic data

	EM-LIFT (*n* = 11)	EL-LIFT *(n* = 11)	*P*-value*
Age (years old [median; IQR])	35 (10; 29–64)	40 (25; 28–65)	0.198
Gender			
Male (*n*; %)	8 (72)	8 (72)	> 0.999
Female (*n*; %)	3 (27)	3 (27)	> 0.999
Ethnicity			
Malay (*n*; %)	8 (72)	9 (81)	> 0.999
Chinese (*n*; %)	3 (27)	2 (18)	> 0.999
Diabetes mellitus (*n*; %)	3 (27)	3 (27)	> 0.999
BMI > 30 (*n*; %)	2 (18)	3 (27)	> 0.999
Steroid use (*n*; %)	1 (9)	5 (45)	> 0.999

Note:

* Fisher’s exact test

**Table 2:** Patients’ operative profiles

	EM-LIFT (*n* = 11)	EL-LIFT (*n* = 11)	*P*-value*
Recurrent presentation	7 (63.63)	5 (45.45)	0.670
Previous surgical intervention	4(36.36)	5 (45.45)	> 0.999
Sepsis	2 (18.18)	0 (0.00)	0.476
Type of fistula			0.361
Simple	4 (36.40)	3 (27.30)	
Complex	7 (63.60)	8 (72.70)	

Notes: Values are presented as number (%);

* Fisher’s exact test

**Table 3:** Comparison of outcomes and postoperative complications

	EM-LIFT (*n* = 11)	EL-LIFT *(n* = 11)	*P*-value
Operative time (minutes)	45 (70.0; 30–110)	60 (45.0; 10–140)	0.970*
Hospital stay (days)	2 (1.0; 1–3 )	0 (1.0; 0–1)	0.001*
Healed wound	10 (90.9)	11 (100.0)	> 0.999^a^
Healing time (months)	2 (1.5; 0.5–4)	2 (3.0; 0.5–4)	0.770*
Recurrence	2 (18.2)	3 (27.3)	0.586*
Recurrence time (months)	5 (0.0; 4–6 )	5 (4.0; 4–6 )	-c
Postoperative complications			
Persistent pain	2 (18)	4 (36)	0.635^a^
Anal fissure	0 (0)	0 (0)	-c
Subcutaneous infection	1 (9)	1 (9)	> 0.999^a^
Local haemorrhage	0 (0)	0 (0)	-c
Faecal incontinence	0 (0)	0 (0)	-c

Notes: Values are presented as number (%); values are presented as median (IQR);

* Mann-Whitney U test;

a Fisher’s exact test;

_c unable to perform statistical comparison between two groups
